# Filament Transport Control for Enhancing Mechanical Properties of Parts Realised by Fused Filament Fabrication

**DOI:** 10.3390/ma15103530

**Published:** 2022-05-14

**Authors:** Arianna Rossi, Giulia Morettini, Michele Moretti, Lorenzo Capponi

**Affiliations:** Industrial Department, University of Perugia, 06125 Perugia, Italy; arianna.rossi2@studenti.unipg.it (A.R.); giulia.morettini@unipg.it (G.M.); lcapponi@illinois.edu (L.C.)

**Keywords:** additive manufacturing, fused filament fabrication, extrusion control, material properties

## Abstract

The fused filament fabrication (FFF) process is widely used for producing prototypes and functional parts for diverse applications. While FFF is particularly attractive due to its cost-effectiveness, on the other hand, the fabricated parts have limitations in terms of large manufacturing time and reduced mechanical properties. The latter is strongly influenced by the fabrication process parameters, which affect the interlayer bonding and the adhesion between consecutive layers. Several works presented in the literature analysed the correlation between mechanical properties and process parameters. It was demonstrated that an increase in the fabrication feed rate causes slippage between filament and the feeding system, which leads to a decrease in the extruded material flow, and thus in part density. This work aims to investigate how the limitation of the slippage phenomenon affects the mechanical properties of parts fabricated using the FFF process. A prototype machine, equipped with a closed-loop control system on filament transport, was used to fabricate samples for tensile tests and dynamical mechanical analysis. Samples fabricated enabling the filament transport control showed an increase both in ultimate tensile strength and elongation at break for those fabricated with disabled control, whilst a decrease in stiffness was observed. In addition, the results showed that the use of a filament transport control system on a FFF machine increases the possibility of fabricating high value-added parts.

## 1. Introduction

**Figure 1:**
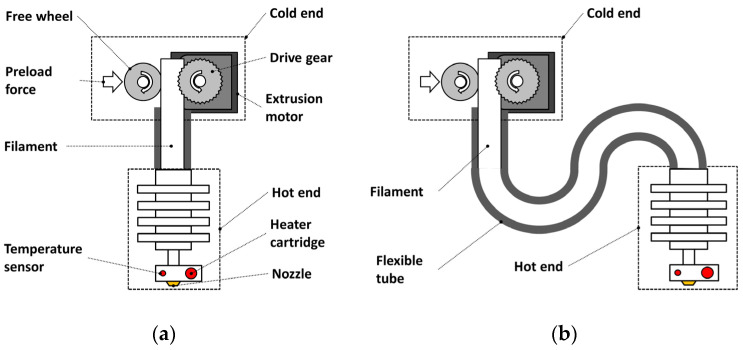
Extruder system layouts: (**a**) Wade extruder (direct) and (**b**) Bowden extruder. Dimensions not to scale.

The fused filament fabrication (FFF) [1,2] process is widely used for producing prototypes and functional parts for various applications [3,4,5,6]. Complex-shaped parts are fabricated using an extrusion system, which moves along a path defined in a part program. With regards to Figure 1, the extrusion system consists of the cold end, where the thermoplastic raw material provided in the form of a filament is pushed towards the hot end, where it is liquefied and expelled through a nozzle. In the cold end, the filament grips onto a motorised gear, while a freewheel pushes the filament against it. In the hot end, a heater cartridge is used as a heat source to liquefy the filament. The extrusion temperature is controlled through a temperature sensor placed close to the nozzle.

The cold end and the hot end can be arranged in two configurations: in the Wade or direct configuration (Figure 1a), the cold end and hot and are merged into a single component, minimising the distance between the motorised gear and the hot end, while in the Bowden configuration (Figure 1b), the cold end and the hot end are separated by a flexible tube.

While the FFF is particularly attractive due to its cost-effectiveness, the fabricated parts have limitations in terms of large manufacturing time and reduced mechanical properties [7]. The latter are strongly influenced by the fabrication process parameters, which, in turn, affect the interlayer bonding and the adhesion between consecutive layers [8,9]. Several works presented in the literature analysed the correlation between mechanical properties and process parameters. It was demonstrated that an increase in the fabrication feed rate causes slippage between filament and the feeding system [10], which leads to a decrease in the extruded material flow [11], and thus in part density [12,13].

To exploit the FFF technology for the fabrication of functional parts, the mechanical characterisation of the fabricated products is needed. In this context, various works have been presented in the literature with the aim of analysing different materials [14], process parameters [15,16] and growth directions [17], highlighting how all these variables affect the mechanical properties of the final product. It has been extensively demonstrated that the growth direction is associated with a lower ultimate tensile strength (UTS) [17], mainly due to a reduced interlayer bonding [8,18,19,20,21,22]. This latter issue has been analysed in [18,23,24], where it is shown how the phenomenon increases with the increase of cooling time and decreases as the temperature difference between the extruded material and the previous layer decreases. In [25,26], the bonding between consecutive layers has been increased by heating the deposition surface with a hot airflow and an infrared laser source, respectively. In [27], the authors defined a correlation between the UTS and the part porosity and density, which, if other parameters are kept constant, decreases when the fabrication feed rate increases [28]. In [29], the authors have demonstrated that, to avoid an excessive reduction of the extruded material flow, and thus a decrease in fabricated parts’ density, the fabrication feed rate should be reduced; however, the solution is opposed to the prospect of increasing the manufacturing speed, considered one of the most relevant topics for all the AM processes [30]. The density reduction observed in [31] is attributable to the slippage phenomenon [10,13], i.e., to the lack of adherence between the motorised gear in the cold end and the filament, which leads to a decrease in the extruded material flow. In [10,11], slippage has been investigated in relation to the compression force exerted on the filament segment comprised between the hot end and the cold end, showing an increase of slippage as the compression force increases. In [11], the authors have shown how slippage increases with the increment of the fabrication feed rate and with the decrement of the extrusion temperature. The slippage phenomenon occurs systematically in FFF systems, although its grade depends on process parameters and on the cold end characteristics [29]. To overcome the issue, usually, slippage is compensated during the generation of the part program by trial-and-error operations [28,32], aimed at defining a multiplicative coefficient for the filament transport, typically referred to as *flow%.* However, this approach has limitations due to the different manufacturing speeds that can be adopted for the same layer, e.g., for the fabrication of walls and infill, and due to variations in extrusion temperature and deposition conditions. These latter affect both contact pressure and extrusion pressure [33], which in turn modify the compression force acting on the filament and hence may cause slippage. Moreover, mechanical and rheological properties of filament also affect the extrusion pressure, and thus slippage [12], making the trial-and-error approach aimed at identifying the best *flow%* value challenging and time-consuming. To overcome such limitations, different closed-loop control systems have been implemented with the aim of controlling the material flow exiting the cold end. In [13], the author has proposed a closed-loop system for filament transport based on imaging, while in [12] the nominal transport of the filament exiting the cold end has been corrected using the value measured with an optical encoder. The last-mentioned closed-loop system acts continuously on the filament feed rate to achieve a minimum difference between the measured transport and the nominal one, as defined in the part program, regardless of process parameters and deposition conditions. Results reported in [12] have shown an increase in the part density as a consequence of the reduction of voids inside the part, and thus an increase of contact regions between deposited strands, both intra- and inter-layer. As slippage is a well-known issue in the literature and it has been proven how it may cause under-extrusion during the process, this work aims to quantitatively investigate its effect on the mechanical properties of FFF parts. For this purpose, a closed-loop system acting on the filament transport was used to minimise the effect of slippage on the flowrate of the extrudate, and parts manufactured with and without the system and using different process parameters were analysed and compared through tensile tests and dynamic mechanical analyses. 

## 2. Materials and Methods

To evaluate the slippage influence on mechanical properties of FFF parts, an experimental campaign was conducted using different values of fabrication feed rate and a closed-loop system capable of compensating slippage. In this way, the correct extrudate flow rate was ensured [12]. A FFF prototype machine [34] was used to fabricate both specimens suitable for tensile tests [35] and dynamic mechanical analysis (DMA) [36]. The FFF machine features a system designed to control the filament transport exiting the cold end. The system is based on a proportional-integrative-derivative (PID) closed-loop controller, here referred to as “CLC”, capable of maintaining the actual filament transport as close as possible to the nominal one (i.e., of compensating slippage), regardless of process parameters and deposition conditions [12]. All the samples were fabricated using the same extrusion temperature, while different values of the fabrication feed rate were adopted. Multiple instances were manufactured for each feed rate value, both with the filament transport control system enabled and disabled (see Section 2.5). All the realised samples shared the same build orientation and positioning. The direction of growth coincided with the direction of the force applied during the tensile test (see Section 2.6). In the literature, the tensile test represents the primary mechanical test for the determination of the material strength properties, as it allows to define the essential engineering for the main design activities, while the DMA is often used to evaluate the viscoelastic properties of polymers. In this work, the first test approach was exploited to determine UTS, elongation at break and Young’s modulus, while the DMA provided measurements of flexural modulus at different frequencies. For each test set-up, the results obtained with the filament transport control system enabled and disabled were compared using one-way ANOVA.

### 2.1. FFF Prototype Machine and Filament Transport Control

**Figure 2:**
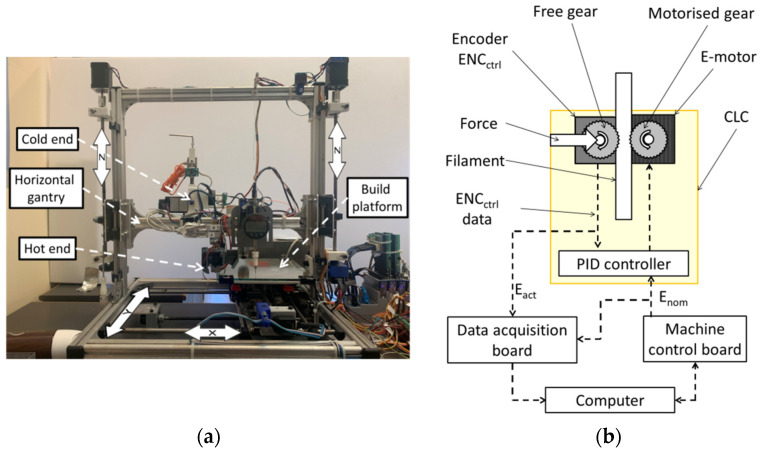
(**a**) The FFF prototype machine and (**b**) the schematics of machine control and data acquisition. Closed-loop control for filament transport is highlighted.

The FFF prototype machine used in this work (Figure 2a) features a cartesian architecture with a build platform moved along the x and y axes by two NEMA17 stepper motors and trapezoidal TRZ8 screws with 8 mm thread. Thanks to two synchronised NEMA 17 stepper motors and TRZ8 trapezoidal screws with 1 mm thread, a horizontal gantry guarantees movement along the z-axis (vertical direction). The maximum achievable fabrication feed rate is 3600 mm/min, with a jerk value equal to 2400 mm/min. The extrusion system has a Bowden configuration, where the hot end and the cold end are mounted on the horizontal gantry. The machine is controlled by a Megatronics 3.2 control board [37] which communicates with a PC via a serial port through the software Repetier Host [38]. The prototype is equipped with heterogeneous sensors [34] acquired by a data acquisition system developed on the LabVIEW^®^ platform [39].

#### Filament Transport Control System

With regards to Figure 2b, the motorised gear inside the cold end, responsible for the filament transport, is driven by a NEMA17 stepper motor, namely the E-motor. A second indented gear, referred to as free-gear, pushes the filament against the driving wheel. The free-gear ensures an adequate compression force on the filament segment comprised between the cold end and the hot end, needed to overcome the dissipative forces (mainly viscous) generated within the hot end. An optical encoder, ${\mathrm{ENC}}_{\mathrm{ctrl}}$, connected to the free-gear, driven exclusively by the filament advancement, allows the measurement of the actual filament transport, $E_{\mathrm{act}}\left(t\right)$, during the manufacturing process at time point t.

A system based on a PID controller, here referred to as CLC (closed-loop controller), acts on the E-motor for minimising the difference between the measured filament transport, $E_{\mathrm{act}}\left(t\right)$, and the nominal value, $E_{\mathrm{nom}}\left(t\right)$. A previous characterisation of the CLC showed the ability of the system to maintain a filament transport error, $\mathrm{err}\left(t\right)=E_{\mathrm{nom}}\left(t\right)-E_{\mathrm{act}}\left(t\right)$, below 0.15 mm under the considered operating conditions (maximum tested feed rate 3600 mm/min). In case of excessive filament transport error, due to a severe slippage which cannot be corrected, the system stops the filament transport and triggers an error signal to the machine control board. A filament transport error threshold was set to 0.3 mm, which is more than double the maximum error observed in previous studies [12].

The $E_{\mathrm{nom}}\left(t\right)$ and ${\mathrm{ENC}}_{\mathrm{ctrl}}$ signals, collected using a LabVIEW^®^-based acquisition system with a sample rate of 100 Hz [34,39], were used to determine slippage, as described in Section 2.2. The CLC system can be manually enabled or disabled; when the CLC is disabled, no corrections are applied to the filament transport.

### 2.2. Slippage Definition

The slippage value, Sl%(t), at time point t is defined as the relative error between the measured filament transport, $E_{\mathrm{act}}\left(t\right)$, and the nominal one, $E_{\mathrm{nom}}\left(t\right)$, calculated referring to the two time points, t and t+Δt, according to Equation (1):

(1) $$\mathrm{Sl}\%\left(t\right)=100*\frac{\left(E_{\mathrm{nom}}\left(t+\Delta t\right)-E_{\mathrm{nom}}\left(t\right)\right)-\left(E_{\mathrm{act}}\left(t+\Delta t\right)-E_{\mathrm{act}}\left(t\right)\right)}{\left(E_{\mathrm{nom}}\left(t+\Delta t\right)-E_{\mathrm{nom}}\left(t\right)\right)}$$

It should be noted that the definition in Equation (1) corresponds to the relative error between the actual transport velocity and the nominal one (i.e., the filament feed rate) when the time interval Δt approaches zero.

Sl%(t) was calculated using Equation (1) with time intervals of Δt=0.01 s (1/sample rate of data acquisition, see Section 2.1). Signals $E_{\mathrm{act}}\left(t\right)$ and $E_{\mathrm{nom}}\left(t\right)$ were pre-processed with a Gaussian filter with σ=0.1 s, for minimising E-motor step-like movement effects.

Since Sl%(t) is time-dependent, while UTS, elongation at break and Young’s modulus are expressed as single values, the mean value of Sl%(t), referred to as $\bar{\mathrm{Sl}\%}$, is used for the analysis. The average value, $\bar{\mathrm{Sl}\%}$, represents a valid performance indicator since the slippage is related to the compression force acting on the filament comprised between the cold end and the hot end, which can be considered constant under steady-state extrusion conditions. Moreover, the filament transport error fluctuation slightly affects the extrudate flowrate, for which the dynamic response of the extrusion system acts as a lowpass filter [11]. Results collected on Sl%(t) showed a limited standard deviation, confirming that the use of $\bar{\mathrm{Sl}\%}$ is reliable for the performed analysis.

### 2.3. Sample Geometry

**Figure 3:**
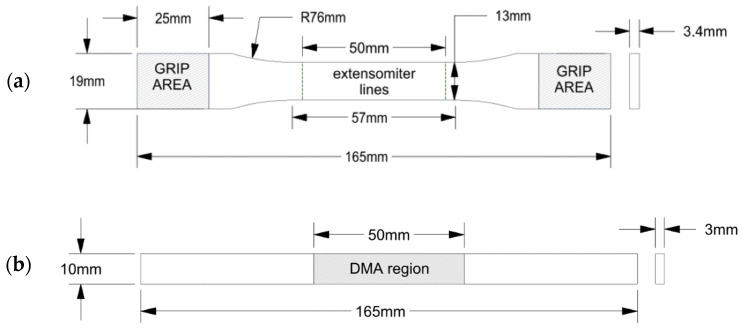
Sample geometries: (**a**) S_TR_ geometry according to the type I of ASTM D638-14 standard, designed for the tensile test, and (**b**) S_DMA_ geometry, according to ASTM D4065-20 standard, designed for the DMA, with tested regions highlighted (see Section 2.9).

The samples for the tensile test, referred to as $S_{\mathrm{TR}}$, were designed under the type I specimen ASTM D638-14 standard, and the reference geometry is depicted in Figure 3a. The geometry of the samples designed for the DMA, referred to as $S_{\mathrm{DMA}}$, follows the ASTM 4065-20 standard indications and it is reported in Figure 3b.

### 2.4. Material

All the samples, $S_{\mathrm{TR}}$ and$\;S_{\mathrm{DMA}}$, were fabricated using a polylactic acid (PLA) filament produced by Fabbrix [40]. The process parameters were chosen according to the ranges suggested by the manufacturer (see Table 1) and adopted in previous studies [12].

### 2.5. Test Plan and Process Parameters

For the fabrication of the $S_{\mathrm{TR}}$ and $S_{\mathrm{DMA}}$ samples, a two-factor DOE with five instances was applied. The two analysed factors were the fabrication feed rate (F) and the CLC state (on/off). Three levels of the fabrication feed rate were selected, corresponding to 1200, 1800 and 2400 mm/min, and the CLC state was associated with two logical levels, 1 or 0, corresponding to state on or off, i.e., samples manufactured with or without enabling the CLC system. 

Temperature and layer height were kept constant and equal to 210 °C and 0.2 mm, respectively. Despite that the CLC was proven to be able to work up to 3600 mm/min [12] and up to 4.8 mm^3^/s at 190 °C (which corresponds to a theoretical fabrication feed rate of 4320 mm/min), the maximum fabrication feed rate adopted was set equal to the jerk of the machine (2400 mm/min). This value was chosen to avoid unsteady-state deposition conditions generated by acceleration and deceleration of the x and y axes while extruding on the short paths of the sample cross-section and to limit the effects of induced vibrations on the samples [29,41]. 

Ten $S_{\mathrm{TR}}$ samples and twenty $S_{\mathrm{DMA}}$ samples were fabricated for each selected feed rate value, divided into an equal number of repetitions with CLC enabled and with CLC disabled, resulting in a total of thirty $S_{\mathrm{TR}}$ samples and sixty $S_{\mathrm{DMA}}$ samples (five repetitions for each factor level combination). The fabrication plan is summarised in Table 2, where the adopted process parameters are listed. 

### 2.6. Sample Orientation and G-Code Preparation

**Figure 4:**
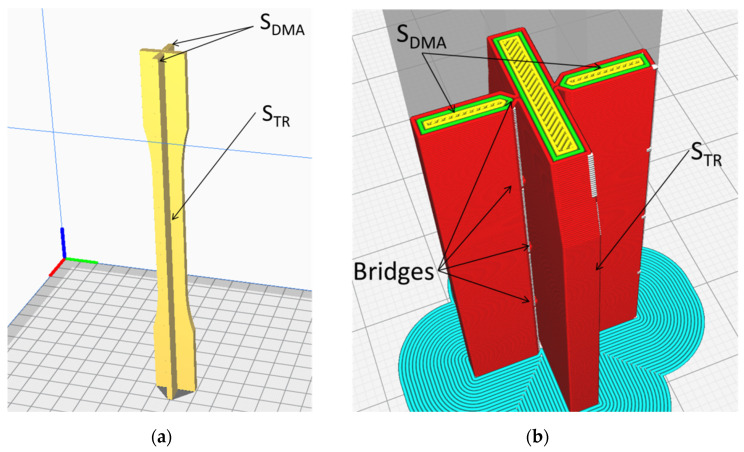
(**a**) Orientation and positioning of S_TR_ and S_DMA_ samples within the building volume and (**b**) detail of the bridge structures.

All the $S_{\mathrm{TR}}$ samples shared the same fabrication position and orientation. The growth direction was chosen parallel to the force direction used in the tensile tests. Previous studies proved that this direction shows the worst mechanical characteristics [20,21]. During each build cycle, two $S_{\mathrm{DMA}}$ samples and one $S_{\mathrm{TR}}$ sample were fabricated, using the layout shown in Figure 4a.$\;S_{\mathrm{DMA}}$ samples were linked to the $S_{\mathrm{TR}}$ sample using bridge structures with a thickness equal to the layer height (0.2 mm), and bridge structures were realised with a periodicity of 10 mm. The detachment of the three parts fabricated during the same process left minimum surface traces; therefore, the effects on the mechanical properties were neglected in this work. 

The configuration depicted in Figure 4 has two purposes: fabricating both sample types at the same time during the same build cycle and limiting the horizontal vibration caused by rapid changes in the velocity of the build platform, which moves in x and y directions (see Section 2.1). All the parts were fabricated using grid infill with −45°/45° raster direction and 100% infill density (see Figure 4b). Part programs were prepared with the Cura^®^ slicer [42].

The estimated manufacturing times were 204, 149 and 123 min for the fabrication feed rates 1200, 1800 and 2400 mm/min, respectively. As the CLC acted only on the filament feed rate, enabling or disabling the CLC did not affect the fabrication time.

### 2.7. Sample Manufacturing, Conditioning and Data Acquisition

The sample manufacturing order was randomised to limit potential bias effects due to environmental conditions. Once the build cycle was completed, the two $S_{\mathrm{DMA}}$ samples were separated from the $S_{\mathrm{TR}}$ sample. All the specimens were labelled and placed into a conditioning chamber with a temperature of 40 °C and ~25% RH. The conditioning time varied from ~192 to ~372 h for the last and first sets of fabricated samples, respectively. 

Slippage was calculated as $\bar{\mathrm{Sl}\%}$ for each build cycle, as described in Section 2.2. In the following sections, slippage values are identified as ${\bar{\mathrm{Sl}\%}}^{i}\left(F,\mathrm{CLC}\right)$, where i stands for the repetition number (i={1…5}, according to Table 2 ), F is the fabrication feed rate (1200, 1800 or 2400 mm/min) and CLC provides information about the CLC state, i.e., on stands for the enabled controller and off for the disabled controller, as summarised in Table 2.

### 2.8. Tensile Test Machine and Test Parameters

An Instron 3382 testing machine [43] was used to perform the tensile tests. All the tests were performed under quasi-static tensile loading with a 2 mm/min displacement rate at a constant room temperature of 25 ± 1 °C. Elongation in the central region of the sample was measured using an extensometer. For each $S_{\mathrm{TR}}$ sample, the UTS value, the elongation at break, $\varepsilon_{\max}$, and the Young’s modulus, $E_{n}$, were calculated.

The following data were calculated for all the $S_{\mathrm{TR}}$ samples: ${\mathrm{UTS}}^{i}\left(F,\mathrm{CLC}\right)$, $\varepsilon_{\max}^{i}\left(F,\mathrm{CLC}\right)$ and $E_{n}^{i}\left(F,\mathrm{CLC}\right)$, where i stands for the repetition number (i={1…5}), F is the fabrication feed rate (1200, 1800 or 2400 mm/min) and CLC is the CLC state (on or off), according to Table 2.

### 2.9. DMA and Test Parameters

The dynamic mechanical analysis (DMA) was performed on $S_{\mathrm{DMA}}$ samples using a Mettler Toledo DMA/STA 1+ machine [44] with a 3-point bending configuration (following the ASTM D5023-15) on a span of 50 mm. The considered area had the same manufacturing vertical coordinate (z) as the region where the extensometer was positioned in the tensile test. 

Tests were performed with an ambient temperature of 25 ± 1 °C with frequencies of between 0.5 and 20 Hz [45] in constant amplitude mode, set to 0.1 mm, as summarised in Table 3. Although the DMA is typically used to investigate the behaviour of materials for the temperature and to estimate the glass transition temperature of thermoplastic polymers, DMA is also widely used to analyse the material performances vs. frequency, which is more interesting for the mechanical characterisation of manufactured parts [46].

The tests on $S_{\mathrm{DMA}}$ samples provided the values $E_{\mathrm{Flex}}^{j}\left(F,\mathrm{Hz},\mathrm{CLC}\right)$, where j is the repetition number (j={1…10}, see Table 2) and Hz is the frequency used for the sample dynamic characterisation. 

### 2.10. Quantitative Assessment of Results

After the tensile tests and the DMA tests, the following data were available for each build cycle:

- ${\bar{\mathrm{Sl}\%}}^{i}\left(F,\mathrm{CLC}\right)$: mean value of slippage (obtained from in-process data)
- ${\mathrm{UTS}}^{i}\left(F,\mathrm{CLC}\right)$: ultimate tensile strength (from tensile test)
- $\varepsilon_{\max}^{i}\left(F,\mathrm{CLC}\right)$: elongation at break (from tensile test)
- $E_{n}^{i}\left(F,\mathrm{CLC}\right)$: Young’s modulus, growth direction (from tensile test)
- $E_{\mathrm{Flex}}^{j}\left(F,\mathrm{Hz},\mathrm{CLC}\right)$: flexural Young’s modulus, perpendicular to growth direction (from DMA).

For each fabrication feed rate, F, ${\bar{\mathrm{Sl}\%}}^{i}\left(F,\mathrm{CLC}\right)$, ${\mathrm{UTS}}^{i}\left(F,\mathrm{CLC}\right)$, $E_{n}^{i}\left(F,\mathrm{CLC}\right)$ and $E_{\mathrm{Flex}}^{j}\left(F,\mathrm{Hz},\mathrm{CLC}\right)$ were analysed using ANOVA and setting a *p*-value threshold equal to 0.05. The two groups compared were those with enabled CLC (CLC=on) and with disabled CLC (CLC=off). The same analysis was performed on $E_{\mathrm{flex}}\left(F,\;\mathrm{CLC},\mathrm{Hz}\right)$ values, where F and Hz were kept constant. 

For each fabrication feed rate and each frequency tested through DMA, the average values, the standard deviation and the relative variation of the average value of the quantities listed above were calculated using Equation (2):

(2) $$\mathrm{Var}\left(\%\right)=100\frac{\mathrm{Data}\left(\mathrm{CLC}=\mathrm{on}\right)-\mathrm{Data}\left(\mathrm{CLC}=\mathrm{off}\right)}{\mathrm{Data}\left(\mathrm{CLC}=\mathrm{off}\right)}$$

where Data is the average value of the considered quantity ($\bar{\mathrm{Sl}\%}$, UTS, $\varepsilon_{\max}$, $E_{N}$ or $E_{\mathrm{flex}}$) obtained from the five test repetitions.

## 3. Results

### 3.1. Slippage

The average values of Sl%(t) ± standard deviation (STD) calculated on each fabrication job are listed in Table 4, while the average values ± standard deviations of $\bar{\mathrm{Sl}\%}\;$are listed in Table 5, gathered according to the fabrication feed rate.

**Figure 5:**
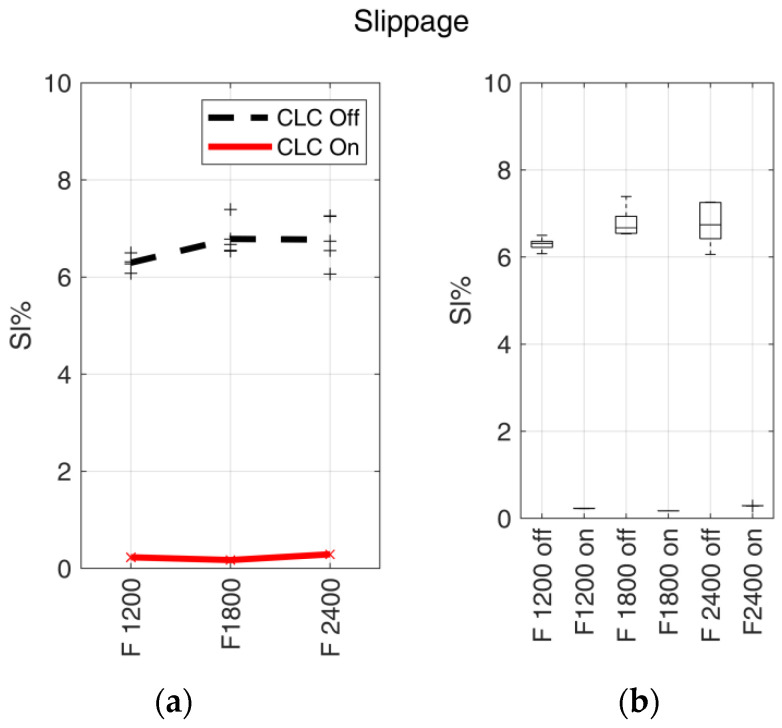
Slippage. (**a**) Slippage trends for the fabrication feed rates (expressed in mm/min). Markers indicate the average slippage value for single tests (${\bar{\mathrm{Sl}\%}}^{i}\left(F,\mathrm{CLC}\right)$), and bold lines indicate the mean values. The continuous red line indicates the samples realised with enabled CLC, and the black dashed line indicates the samples realised with disabled CLC. (**b**) Box plots of $\bar{\mathrm{Sl}\%}$ vs. filament feed rate, F, with enabled (on) and disabled (off) CLC.

$\bar{\mathrm{Sl}\%}$ values are reported in Figure 5 in relation to the fabrication feed rate, both for enabled and disabled CLC.

### 3.2. Tensile Test

**Figure 6:**
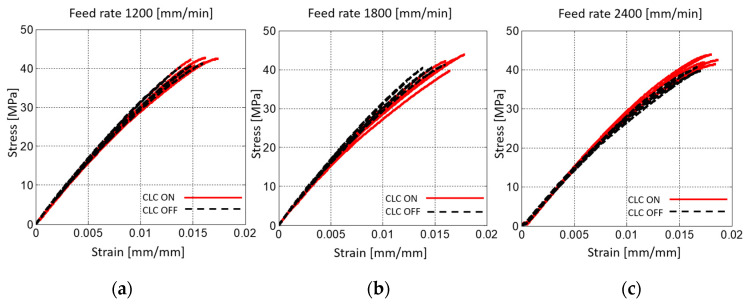
Stress–strain plots for S_TR_ samples fabricated with (**a**) F = 1200 mm/min, (**b**) F = 1800 mm/min and (**c**) F = 2400 mm/min. Black dashed lines and continuous red lines represent samples fabricated with disabled CLC and enabled CLC, respectively.

Figure 6 depicts the stress–strain curves obtained from the tensile tests. The results are gathered in three subplots according to the feed rate used to fabricate the correspondent samples.

#### 3.2.1. UTS

**Figure 7:**
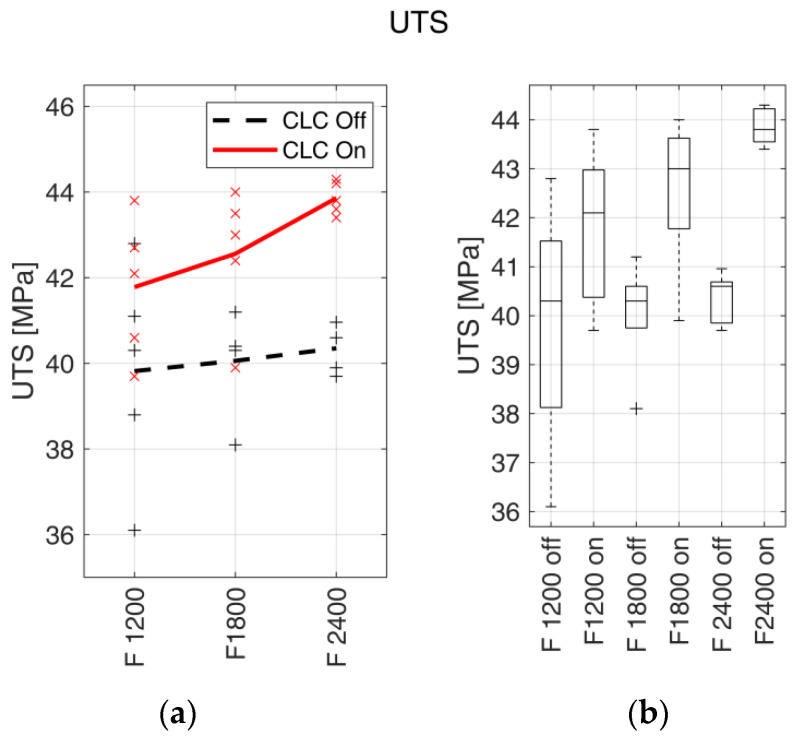
UTS. (**a**) UTS trends with respect to the fabrication feed rates (expressed in mm/min). Markers indicate the measured values for each test, and bold lines indicate the mean values. The continuous red line indicates the samples realised with enabled CLC, and the black dashed line indicates the samples realised with disabled CLC. (**b**) Box plots of UTS vs. filament feed rate with enabled (on) and disabled (off) CLC.

The UTS trends for the fabrication feed rate are reported in Figure 7a, with the respective boxplots (Figure 7b). The correspondent numerical results (average values ± standard deviation, percentage variation and *p*-values) are reported in Table 6. 

#### 3.2.2. Elongation at Break

In Table 7, the elongation at break ($\varepsilon_{\max}$) average values ± standard deviation, the percentage variation and the correspondent *p*-values concerning the fabrication feed rates are reported. 

**Figure 8:**
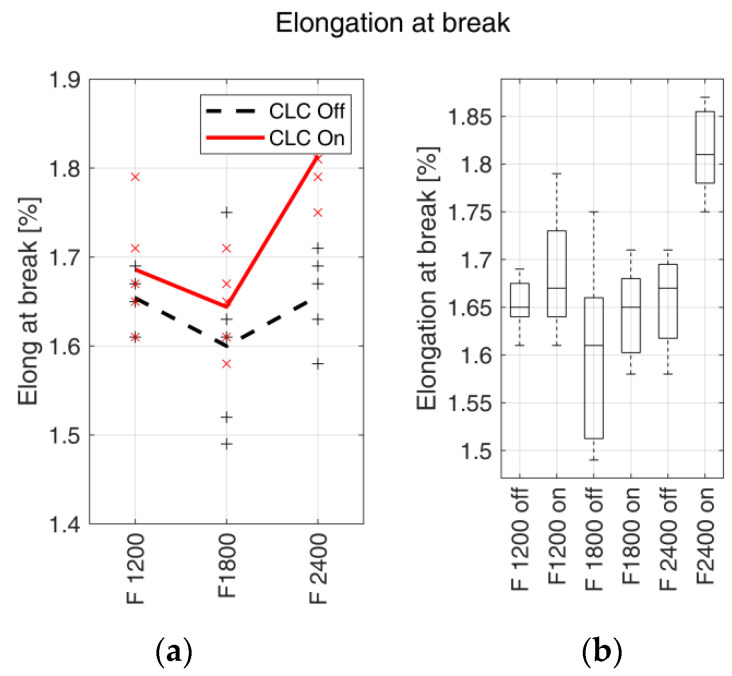
Elongation at break. (**a**) $\varepsilon_{\max}$trends with respect to the fabrication feed rates (expressed in mm/min). Markers indicate the measured values for each test, and bold lines indicate the mean values. The continuous red line indicates the samples realised with enabled CLC, and the black dashed line indicates the samples realised with disabled CLC. (**b**) Box plots of $\varepsilon_{\max}$ vs. filament feed rate with enabled (on) and disabled (off) CLC.

The trends of $\varepsilon_{\max}$ in relation to the feed rates are shown in Figure 8 with the relative boxplots.

#### 3.2.3. Young’s Modulus

The average values ± standard deviation, the percentage variation of $E_{N}$ and the correspondent *p*-values are reported in Table 8. 

**Figure 9:**
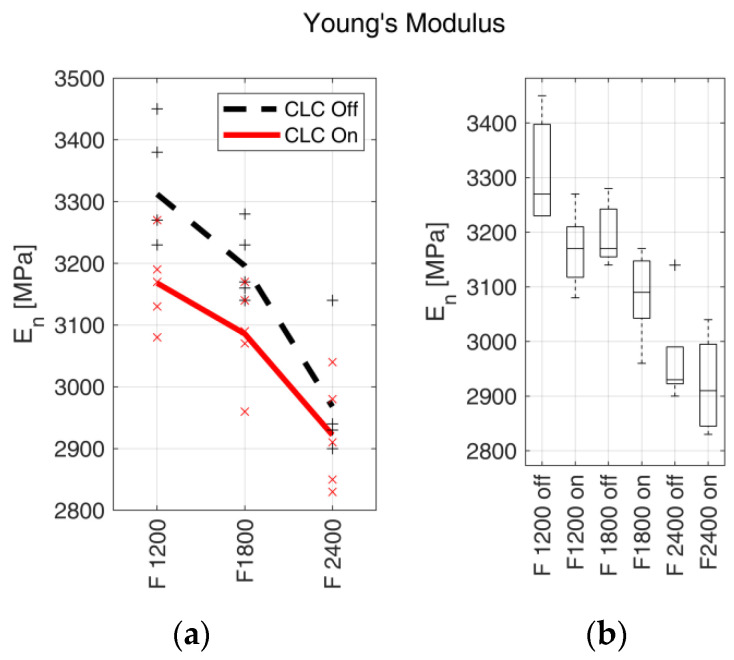
Young’s modulus $E_{N}$. (**a**) $E_{N}$ trends with respect to the fabrication feed rates (expressed in mm/min). Markers indicate the measured values for each test, and bold lines indicate the mean values. The continuous red line indicates the samples realised with enabled CLC, and the black dashed line indicates the samples realised with disabled CLC. (**b**) Box plots of $E_{N}$ vs. filament feed rate with enabled (on) and disabled (off) CLC.

The trends of $E_{n}$ in relation to the feed rates are shown in Figure 9 with the relative boxplots.

### 3.3. Dynamic Mechanical Analysis

Table 9 summarises the average values ± standard deviation, the percentage variations of $E_{\mathrm{Flex}}^{j}$ and the *p*-values, and data are divided according to analysed frequencies. 

**Figure 10:**
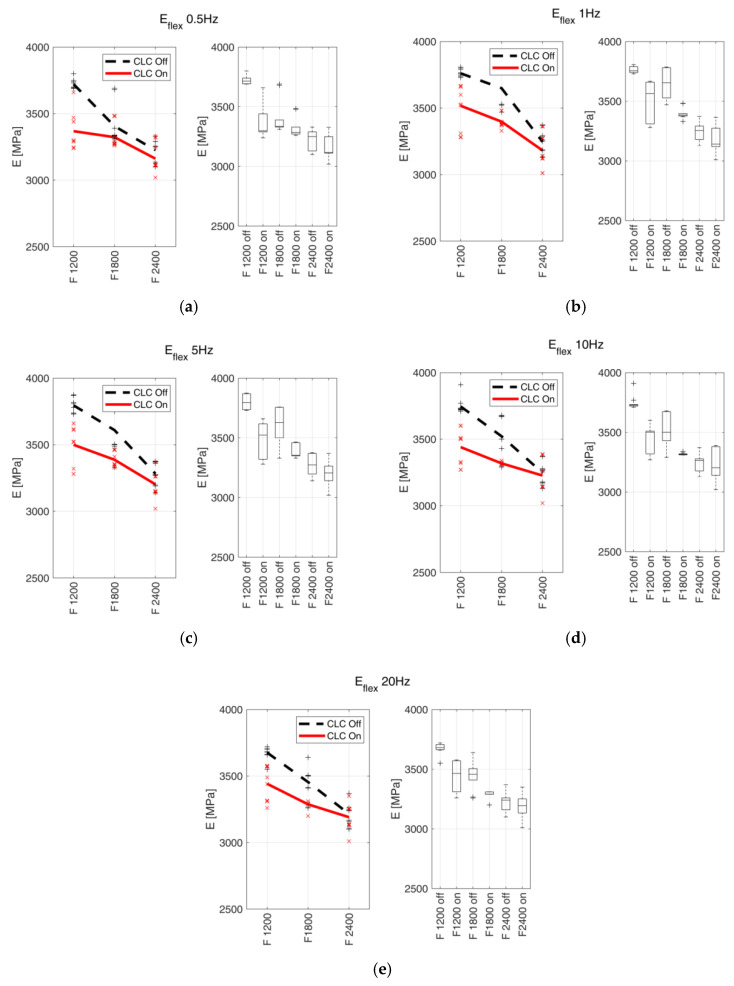
E_flex_ measured through DMA and relative boxplots at (**a**) 0.5 Hz, (**b**) 1 Hz, (**c**) 5 Hz, (**d**) 10 Hz and (**e**) 20 Hz. Markers indicate the measured values, and bold lines indicate the mean values. The continuous red line indicates the samples realised with enabled CLC, and the black dashed line indicates the samples realised with disabled CLC.

For each tested frequency, trends and box plots of $E_{\mathrm{flex}}$ are reported in Figure 10.

## 4. Discussion

### 4.1. Slippage

Average values of Sl%(t) calculated on each fabrication process exhibited a low standard deviation, and thus the use of $\bar{\mathrm{Sl}\%}$ as a global performance indicator did not introduce significant uncertainty.

Trends of $\bar{\mathrm{Sl}\%}$ values showed how the CLC can substantially reduce the filament transport error caused by slippage. Results were congruent with those obtained in previous work [12] within the fabrication feed rate range from 1200 to 2400 mm/min. The decrease of $\bar{\mathrm{Sl}\%}$ was visible in Figure 5, where slippage went from values above 6% (disabled CLC) to values lower than 0.3% (enabled CLC). Moreover, the standard deviation of $\bar{\mathrm{Sl}\%}$ values was significantly reduced with enabled CLC, as shown in Figure 5b, demonstrating increased extrusion process control. *p*-values lower than the threshold highlight the strong statistical separation between the analysed groups (CLC disabled and CLC enabled). Considering the value of $\bar{\mathrm{Sl}\%}$ calculated on samples fabricated, respectively, with CLC enabled and disabled, a slight variation was observed concerning the feed rate, and therefore it can be considered to have an influence on slippage in the analysed scenario.

### 4.2. Tensile Test

#### 4.2.1. UTS

UTS average values increased with the increase of the fabrication feed rate, F, both with enabled and disabled CLC. In [9,18,24], the authors showed how interlayer bonding depends on the temperature difference between the previous layer and the newly deposited material, but also on the cooling rate. Both the phenomena indeed acted on the mutual material diffusion between newly extruded and previously deposited material at the interface. Therefore, the observed behaviour is explainable as a consequence of the different temperature fields encountered by the material during the deposition [18]. As the build cycles have a different duration (see Section 2.6), the parts fabricated with the higher feed rate had less time for the layer cooling and, consequently, the temperature difference between the extruded material and the underlying layer was lower. As reported in Section 4.1, $\bar{\mathrm{Sl}\%}$ slightly changed with the feed rate, and thus the increase of UTS with the feed rate can be exclusively considered as an effect of the temperature field.

A comparison between the UTS of parts fabricated with the same feed rate demonstrated how the UTS average values were always higher with CLC enabled, for all the analysed feed rates. The average UTS value increased from 4.97% with a 1200 mm/min feed rate to 8.70% for the 2400 mm/min feed rate, with an intermediate value of 6.24% at 1800 mm/min. The increase of UTS can be interpreted under two aspects, both related to the increased flow of extruded material in the case of enabled CLC (as it compensates for the slippage, which, as previously reported, determines a decrease in the extruded material flow). The first aspect, related to thermal phenomena, is that a larger flow of material exiting the nozzle leads to an increase in the thermal capacity of the deposited material, i.e., of the layer being manufactured, and consequentially of the whole fabricated part. Considering the thermal exchange under constant environmental conditions, the increase in the thermal capacity of the previously fabricated layers resulted in a decrease in the cooling gradient. Consequently, the temperature difference between the extruded material and the deposition surface was reduced. The result was an increase in the interlayer bonding [9]. The second aspect consists in the higher filling of parts realised with enabled CLC, as described in [12], where the cross-section of parts realised with enabled CLC with the same material and process parameters adopted in this work showed a substantial reduction in void number and extension. In this case, the deposited layers featured a larger contact surface due to the reduction of voids inside the fabricated part. Moreover, the voids are typically considered stress concentration regions and, consequently, crack propagation trigger points. From the data analysis viewpoint, it can be observed that the *p*-values tended to decrease as the manufacturing speed increased, showing an augmented significance of the results for the manufacturing speed. Concerning the parts fabricated using the feed rate of 1200 mm/min, a *p*-value larger than the established threshold was obtained, and thus the augmentation in part density achieved through the adoption of the filament transport control was less significant at lower fabrication feed rates.

#### 4.2.2. Elongation at Break

In terms of elongation at break, there was an overall increase in samples manufactured with CLC enabled. This is consistent with previous studies, where an increase in the interlayer air gaps was associated with the decrease of the elongation at break [47,48]. Higher relative variation (11.8%) was found at the maximum analysed fabrication feed rate, whilst the minimum value of 1.93% was obtained at the minimum fabrication feed rate. A local minimum was observed on samples fabricated at a feed rate equal to 1800 mm/min for both sample groups, manufactured with CLC enabled and CLC disabled. The distributions are shown in Figure 8b, highlighting an overlap at fabrication feed rates equal to 1200 and 1800 mm/min, whilst a strong separation was noticeable at 2400 mm/min. This suggests that the filament transport controller had a considerable effect on elongation at break at higher fabrication feed rates, which is supported by the corresponding *p*-values.

#### 4.2.3. Elasticity

Concerning the stiffness, $E_{N}$, the trend was opposed to the ones of UTS and elongation at break. $E_{N}$ indeed tended to decrease when the fabrication feed rate increased, both with enabled and disabled CLC. It was also possible to observe how, using the same feed rate, $E_{N}$ was higher in samples realised with disabled CLC, while the relative difference, Var(%), decreased when the feed rate was increased. Additionally, in this case, to analyse the trend, it is helpful to resort to what was previously introduced about cooling thermal gradients and part filling. Considering the two $E_{N}$ trends separately, a higher fabrication feed rate led to a decrease in the thermal gradient between the extruded material and the previous layer, which resulted in a longer cooling time. The increase in cooling time affected the recrystallisation of the thermoplastic polymer during the solidification phase, leading to lower stiffness. Similar results were obtained in [25], where hot air was dispensed on the fabricated layer before it was covered by the next layer. In [25], an increase of UTS and a decrease of stiffness were observed, exclusively addressed by thermal effects. On the other hand, other studies [20,49] suggest that the behaviour of $E_{N}$ can be considered material-dependent and primarily correlated to material properties.

By analysing the effect of CLC, the decrease in $E_{N}$ with enabled CLC, for all the manufacturing feed rates, can be attributed to the increased heat capacity due to the greater mass of deposited material, which led to a decrease in the thermal gradient (as also mentioned in Section 4.2.1). On the other hand, an increase in the extruded material flow with enabled CLC led to an increase in the effective cross-sectional area, the macroscopic effect of which is expected to be an increase in $E_{N}$ [48,50]. In this case, the two effects are in contrast, and the decrease of $E_{N}$ (when the fabrication feed rate increases and with enabled CLC) is due to the prevalence of the thermal effects over the increase in the resistant cross-section. 

From a statistical standpoint, the data collected for the higher speeds presented a *p*-value larger than the established threshold and average values with a relative difference of about 1.5% (see Table 8). This shows that the CLC effect was marginal at higher fabrication feed rates, making the two $E_{N}$ distributions well-overlapped. 

### 4.3. DMA

The results obtained from the DMA followed the same trends obtained with the tensile test (see Section 4.2.3). The decreasing trends of $E_{flex}$ confirmed the ones of $E_{N}$, with $E_{flex}$ decreasing when the fabrication feed rate increased. The relation $E_{flex}\left(CLC=on\right)<E_{flex}\left(CLC=off\right)$ was valid in all the tested conditions, regardless of the applied load frequency. For all the load frequencies analysed, the average values tended to converge. Regarding the statistical analysis, the groups of data collected from samples fabricated with the feed rates equal to 1200 and 1800 mm/min respected the condition *p*-value < 0.05, except for the 0.5 Hz frequency, where one value over the set threshold was observed. On the other hand, all datasets referring to the manufacturing feed rate of 2400 mm/min did not meet the *p*-value < 0.05 condition. In this last case, the two data dispersions were overlapping, and thus the CLC effect on the $E_{\mathrm{flex}}$ values became less significant. It can be observed that both $E_{\mathrm{flex}}$ and $E_{N}$ show decreasing trends with respect to feed rates, as discussed in Section 4.2.3.

## 5. Conclusions

This work assessed the mechanical properties of samples fabricated through FFF using tensile tests and dynamical mechanical analysis. Tested samples were produced using a prototype machine equipped with a closed-loop control system acting on filament transport. The CLC can actively control the under-extrusion phenomena caused by slippage between the filament and the motorised gear within the cold end. The results clearly showed a significant overall improvement of UTS (up to 8.7%) and elongation at break (up to 11.8%) in samples realised with the filament transport control enabled. Conversely, stiffnesses from tensile tests and dynamical mechanical analysis showed an opposite trend: samples realised with the filament transport control disabled showed a higher stiffness than samples realised with enabled CLC. 

Finally, the outcomes of the experimental tests proved that increasing the fabrication feed rate corresponds to an increase in the UTS and elongation at break values. Relying on the observed results, higher fabrication feed rates can be adopted to fabricate parts without compromising their mechanical properties. Further analysis needs to be carried out in order to assess the mechanical properties of parts realised with different process parameters, different materials and higher feed rates, where slippage becomes more severe. 

## Figures and Tables

**Table 1:** PLA filament characteristics.

Filament Specifications and Print Settings	
Diameter	1.75 ± 0.05 mm
Maximum roundness deviation	2%
Print temperature (T)	200–215 °C
Fabrication feed rate (F)	40–80 mm/s

**Table 2:** DOE summary.

Test ID	Temperature (°C)	F (mm/min)	Layer Height (mm)	Flow Control (ON/OFF)	Number of Samples S_TR_	Number of Samples S_DMA_
1	210	1200	0.2	OFF	5	10
2	210	1200	0.2	ON	5	10
3	210	1800	0.2	OFF	5	10
4	210	1800	0.2	ON	5	10
5	210	2400	0.2	OFF	5	10
6	210	2400	0.2	ON	5	10

**Table 3:** Parameters adopted for the dynamic characterisation of S_DMA_ samples.

Sample #	1	2	3	4	5
Frequency (Hz)	0.5	1	5	10	20
Temperature (°C)	25	25	25	25	25
Span (mm)	50	50	50	50	50
Constant amplitude (mm)	0.1	0.1	0.1	0.1	0.1

**Table 4:** Average values of Sl%(t) and standard deviation (STD) calculated on each fabrication job.

Sl%(t)		Sample Number	CLC off	CLC on
Fabrication Feed rate (mm/min)	1200	1	6.3128 ± 0.159	0.2298 ± 0.0038
		2	6.078 ± 0.173	0.2336 ± 0.0036
		3	6.499 ± 0.141	0.2283 ± 0.0045
		4	6.3163 ± 0.161	0.2246 ± 0.0032
		5	6.27 ± 0.175	0.2347 ± 0.0047
	1800	1	6.6691 ± 0.315	0.1718 ± 0.0017
		2	6.5481 ± 0.296	0.1746 ± 0.0026
		3	6.5327 ± 0.358	0.1716 ± 0.0013
		4	6.7829 ± 0.327	0.1722 ± 0.0019
		5	7.39 ± 0.368	0.1761 ± 0.0022
	2400	1	6.060 ± 0.496	0.2867 ± 0.0028
		2	6.7412 ± 0.538	0.293 ± 0.0032
		3	6.5426 ± 0.621	0.2929 ± 0.0032
		4	7.26 ± 0.687	0.2935 ± 0.0021
		5	7.25 ± 0.602	0.2921 ± 0.0029

**Table 5:** Mean values of slippage.

$\bar{Sl\%}$		CLC off	CLC on	Var (%)	*p*-Value
Fabrication Feed rate (mm/min)	1200	6.2952 ± 0.1505	0.2302 ± 0.0041	−96.34	2.6 × 10^−13^
	1800	6.7846 ± 0.3533	0.1733 ± 0.002	−97.45	1.17 × 10^−10^
	2400	6.7708 ± 0.5067	0.2916 ± 0.0028	−95.69	2.42 × 10^−9^

**Table 6:** UTS values.

UTS (MPa)		CLC off	CLC on	Var (%)	*p*-Value
Fabrication feed rate (mm/min)	1200	39.82 ± 2.5310	41.78 ± 1.6392	4.97	0.1821
	1800	40.06 ± 1.1589	42.56 ± 1.601	6.24	0.0222
	2400	40.35 ± 0.5294	43.86 ± 0.3847	8.70	2.16 × 10^−6^

**Table 7:** Elongation at break.

$\varepsilon_{\max}\left(\%\right)$		CLC off	CLC on	Var (%)	*p*-Value
Fabrication feed rate (mm/min)	1200	1.6540 ± 0.0297	1.6860 ± 0.0684	1.93	0.3653
	1800	1.6002 ± 0.1025	1.6444 ± 0.0518	2.74	0.4147
	2400	1.6560 ± 0.0518	1.8514 ± 0.0477	11.8	1 × 10^−3^

**Table 8:** Young’s modulus E_N_.

E_N_ (MPa)		CLC off	CLC on	Var (%)	*p*-Value
Fabrication feed rate (mm/min)	1200	3312 ± 98.59	3168 ± 70.85	−4.35	0.0292
	1800	3196 ± 57.70	3086 ± 80.81	−3.44	0.0383
	2400	2968 ± 97.31	2922 ± 88.14	−1.55	0.4560

**Table 9:** Numerical results obtained from dynamic mechanical analysis.

Fabrication Feed Rate (mm/min)	CLC off	CLC on	Var %	*p*-Value
	Eflex (MPa)@0.5 Hz			
1200	3721 ± 35.62	3368 ± 132.44	−9.48	1.9 × 10^−7^
1800	3406 ± 148.67	3323 ± 86.14	−2.44	0.1454
2400	3224 ± 83.22	3161 ± 101.99	−1.95	0.1475
	**Eflex (MPa)@1 Hz**			
1200	3761 ± 28.64	3517 ± 165.57	−6.49	2.3 × 10^−4^
1800	3649 ± 142.09	3398 ± 47.73	−6.88	4.86 × 10^−5^
2400	3246 ± 88.30	3182 ± 129.44	−1.97	0.2094
	**Eflex (MPa)@5 Hz**			
1200	3796 ± 61.17	3499 ± 141.94	−7.82	2 × 10^−5^
1800	3610 ± 160.49	3387 ± 55.92	−6.18	6.18 × 10^−4^
2400	3283 ± 86.21	3202 ± 99.13	−2.46	0.0691
	**Eflex (MPa)@10 Hz**			
1200	3746 ± 60.01	3441 ± 129.99	−8.14	2.53 × 10^−6^
1800	3522 ± 151.51	3183 ± 9.44	−9.62	4.96 × 10^−4^
2400	3247 ± 83.06	3227 ± 128.88	−6.16	0.6789
	**Eflex (MPa)@20 Hz**			
1200	3676 ± 49.2	3442 ± 131.27	−6.37	5.06 × 10^−5^
1800	3455 ± 130.97	3288 ± 32.15	−4.83	1 × 10^−3^
2400	3215 ± 81.92	3191 ± 97.45	−7.46	0.555

## References

[1] Vyavahare S., Teraiya S., Panghal D., Kumar S. (2020). Fused deposition modelling: A review. Rapid Prototyp. J..

[2] Gibson I., Rosen D.W., Stucker B. (2014). Additive Manufacturing Technologies.

[3] Youssef A., Hollister S.J., Dalton P.D. (2017). Additive manufacturing of polymer melts for implantable medical devices and scaffolds. Biofabrication.

[4] Maurizi M., Slavič J., Cianetti F., Jerman M., Valentinčič J., Lebar A., Boltežar M. (2019). Dynamic measurements using FDM 3D-printed embedded strain sensors. Sensors.

[5] Jaksic N.I., Desai P.D. (2019). Characterization of 3D-printed capacitors created by fused filament fabrication using electrically-conductive filament. Procedia Manuf..

[6] Ghomi E.R., Eshkalak S.K., Singh S., Chinnappan A., Ramakrishna S., Narayan R. (2021). Fused filament printing of specialized biomedical devices: A state-of-the art review of technological feasibilities with PEEK. Rapid Prototyp. J..

[7] Koch C., van Hulle L., Rudolph N. (2017). Investigation of mechanical anisotropy of the fused filament fabrication process via customized tool path generation. Addit. Manuf..

[8] Li H., Wang T., Sun J., Yu Z. (2018). The effect of process parameters in fused deposition modelling on bonding degree and mechanical properties. Rapid Prototyp. J..

[9] Vanaei H.R., Raissi K., Deligant M., Shirinbayan M., Fitoussi J., Khelladi S., Tcharkhtchi A. (2020). Toward the understanding of temperature effect on bonding strength, dimensions and geometry of 3D-printed parts. J. Mater. Sci..

[10] Nienhaus V., Spiehl D., Dörsam E. (2021). Investigations on roller-based filament drives. J. Manuf. Mater. Process..

[11] Moretti M., Rossi A., Senin N. (2021). In-process simulation of the extrusion to support optimisation and real-time monitoring in fused filament fabrication. Addit. Manuf..

[12] Moretti M., Rossi A. (2021). Closed loop filament feed control in fused filament fabrication. 3D Print. Addit. Manuf..

[13] Greeff G.P., Schilling M. (2017). Closed loop control of slippage during filament transport in molten material extrusion. Addit. Manuf..

[14] Dey A., Eagle I.N.R., Yodo N. (2021). A review on filament materials for fused filament fabrication. J. Manuf. Mater. Process..

[15] Khan S., Joshi K., Deshmukh S. (2022). A comprehensive review on effect of printing parameters on mechanical properties of FDM printed parts. Mater. Today Proc..

[16] Harris M., Potgieter J., Archer R., Arif K.M. (2019). Effect of material and process specific factors on the strength of printed parts in fused filament fabrication: A review of recent developments. Materials.

[17] Corapi D., Morettini G., Pascoletti G., Zitelli C. (2019). Characterization of a polylactic acid (PLA) produced by Fused Deposition Modeling (FDM) technology. Procedia Struct. Integr..

[18] Sun Q., Rizvi G.M., Bellehumeur C.T., Gu P. (2008). Effect of processing conditions on the bonding quality of FDM polymer filaments. Rapid Prototyp. J..

[19] Coogan T.J., Kazmer D.O. (2019). Modeling of interlayer contact and contact pressure during fused filament fabrication. J. Rheol..

[20] Chacón J.M., Caminero M.A., García-Plaza E., Núñez P.J. (2017). Additive manufacturing of PLA structures using fused deposition modelling: Effect of process parameters on mechanical properties and their optimal selection. Mater. Des..

[21] Durgun I., Ertan R. (2014). Experimental investigation of FDM process for improvement of mechanical properties and production cost. Rapid Prototyp. J..

[22] Gordelier T.J., Thies P.R., Turner L., Johanning L. (2019). Optimising the FDM additive manufacturing process to achieve maximum tensile strength: A state-of-the-art review. Rapid Prototyp. J..

[23] Pollard D., Ward C., Herrmann G., Etches J. (2017). Filament Temperature Dynamics in Fused Deposition Modelling and Outlook for Control. Procedia Manuf..

[24] Kuznetsov V.E., Solonin A.N., Tavitov A., Urzhumtsev O., Vakulik A. (2020). Increasing strength of FFF three-dimensional printed parts by influencing on temperature-related parameters of the process. Rapid Prototyp. J..

[25] Prajapati H., Salvi S.S., Ravoori D., Qasaimeh M., Adnan A., Jain A. (2021). Improved print quality in fused filament fabrication through localized dispensing of hot air around the deposited filament. Addit. Manuf..

[26] Du J., Wei Z., Wang X., Wang J., Chen Z. (2016). An improved fused deposition modeling process for forming large-size thin-walled parts. J. Mater. Process. Technol..

[27] Zekavat A.R., Jansson A., Larsson J., Pejryd L. (2019). Investigating the effect of fabrication temperature on mechanical properties of fused deposition modeling parts using X-ray computed tomography. Int. J. Adv. Manuf. Technol..

[28] Duddleston L.J.L., Woznick K., Koch C., Capote G.M., Rudolph N., Osswald T.A. (2017). Extrudate mass flow rate analysis in Fused Filament Fabrication (FFF): A cursory investigation of the effects of printer parameters. Annu. Tech. Conf.—ANTEC Conf. Proc..

[29] Go J., Schiffres S.N., Stevens A.G., Hart A.J. (2017). Rate limits of additive manufacturing by fused filament fabrication and guidelines for high-throughput system design. Addit. Manuf..

[30] Singh S., Singh G., Prakash C., Ramakrishna S. (2020). Current status and future directions of fused filament fabrication. J. Manuf. Process..

[31] Santana L., Ahrens C.H., Da Costa Sabino Netto A., Bonin C. (2017). Evaluating the deposition quality of parts produced by an open-source 3D printer. Rapid Prototyp. J..

[32] Loh G.H., Pei E., Gonzalez-Gutierrez J., Monzón M. (2020). An overview of material extrusion troubleshooting. Appl. Sci..

[33] Kazmer D.O., Colon A.R., Peterson A.M., Kim S.K. (2021). Concurrent characterization of compressibility and viscosity in extrusion-based additive manufacturing of acrylonitrile butadiene styrene with fault diagnoses. Addit. Manuf..

[34] Moretti M., Bianchi F., Senin N. (2020). Towards the development of a smart Fused Filament Fabrication system using multi-sensor data fusion for in-process monitoring. Rapid Prototyp. J..

[35] ASTM (2014). ASTM D638-14: Standard Test Method for Tensile Properties of Plastics. https://www.astm.org/Standards/D638.

[36] Kevin N.M., Menard P. (2020). Dynamic Mechanical Analysis.

[37] Meijer B. (2018). Megatronics v3.2 Datasheet, 2018. https://reprapworld.com/datasheets/datasheetmegatronicsv32.pdf.

[38] Repetier (2018). Software Repetier Host. https://www.repetier.com/.

[39] National Instruments. Labview. https://www.ni.com/en-us/shop/labview/labview-details.html.

[40] Fabbrix (2019). Fabbrix Materials. https://www.fabbrix.com/fabbrix-materials.

[41] Ertay D.S., Yuen A., Altintas Y. (2018). Synchronized material deposition rate control with path velocity on fused filament fabrication machines. Addit. Manuf..

[42] Ultimaker (2019). Ultimaker Cura. https://ultimaker.com/software/ultimaker-cura.

[43] Instron (2021). Instron, Mechanical Testing. https://instron.com/en/.

[44] Mettler Toledo, Dynamic Mechanical Analyzer (DMA) DMA/SDTA 1+. https://www.mt.com/ch/en/home/products/Laboratory_Analytics_Browse/TA_Family_Browse/DMA/dmasdta1.html.

[45] Morettini G., Palmieri M., Capponi L., Landi L. (2021). Experimental determination of mechanical properties of PLA FDM 3d-printed structures for dynamic analysis simulation. Prog. Addit. Manuf..

[46] Palmieri M., Zucca G., Morettini G., Landi L., Cianetti F. (2022). Vibration Fatigue of FDM 3D Printed Structures: The Use of Frequency Domain Approach. Materials.

[47] Tian X., Liu T., Yang C., Wang Q., Li D. (2016). Interface and performance of 3D printed continuous carbon fiber reinforced PLA composites. Compos. Part A Appl. Sci. Manuf..

[48] Wang S., Ma Y., Deng Z., Zhang S., Cai J. (2020). Effects of fused deposition modeling process parameters on tensile, dynamic mechanical properties of 3D printed polylactic acid materials. Polym. Test..

[49] Naveed N. (2021). Investigating the material properties and microstructural changes of fused filament fabricated PLA and tough-PLA parts. Polymers.

[50] Quintana J.L.C., Redmann A., Capote G.A.M., Pérez-Irizarry A., Bechara A., Osswald T.A., Lakes R. (2019). Viscoelastic properties of fused filament fabrication parts. Addit. Manuf..

